# Efficacy of memory training in healthy community-dwelling older people: study protocol for a randomized controlled trial

**DOI:** 10.1186/s12877-015-0110-4

**Published:** 2015-10-01

**Authors:** Anna Pérez, Marta Roqué, Sara Domènech, Rosa Monteserín, Núria Soriano, Xavier Blancafort, Maria Bosom, Cristina Vidal, Montse Petit, Núria Hortal, Carles Gil, Albert Espelt, Maria José López

**Affiliations:** Agència de Salut Púbica de Barcelona (ASPB), Pl Lesseps, Barcelona, 1 (08023) Spain; Ciber de Epiedmiología y Salud Pública (CIBERESP), C/ Melchor Fernández, Almagro, Madrid 3-5 (28029) Spain; Institut d’Investigació Biomèdica (IIB Sant Pau), C/ Sant Antoni Maria Claret, Barcelona, 167 (08025) Spain; Centro Cochrane Iberoamericano, C/ Sant Antoni Maria Claret, Barcelona, 167 (08025) Spain; Institut de l’Envelliment, C/ Sant Antoni Maria Claret, Barcelona, 171 (08041) Spain; Equip d’Atenció Primària Sardenya, C/ de Sardenya, Barcelona, 466 (08025) Spain; Centre d’Atenció Primària Roquetes-Canteres, C/ Garigliano, Barcelona, 23 (08042) Spain; Centre d’Atenció Primària Sant Rafael, Pg. de la Vall d’Hebron, Barcelona, 107-117 (08035) Spain; Fundació Pere Tarrés, C/ Numància, Barcelona, 149-151 (08029) Spain; Departament de Gent Gran Ajuntament de Barcelona, C/ València, Barcelona, 344 (08009) Spain; Departament de Ciències Experimentals i de la Salut, Universitat Pompeu Fabra (UPF) (Experimental and Health Sciences Department, Pompeu Fabra University, Barcelona (Spain)), C/ Doctor Aiguader, Barcelona, 88 (08003) Spain

**Keywords:** Memory training, Randomized control trial, Older people, Rivermead behavioural memory test, Health-related quality of life

## Abstract

**Background:**

There is limited evidence on the efficacy and social utility of cognitive training. To address this, we have designed a randomized controlled trial to assess the effectiveness of memory training workshops for healthy older people in terms of their short- and long-term impact on cognitive function, health-related quality of life, and functionality.

**Methods/design:**

A randomized controlled trial will be performed in health care centers in Barcelona (Spain) through comparison of a group of individuals participating in memory training workshops (experimental group) with another group with similar characteristics not participating in the workshops (control group). The intervention will consist of twelve 90-minute group sessions imparted once a week by a psychologist specialized in memory training. The groups will each comprise approximately 15 people, for a total number of 230 patients involved in the study. Each session has its own objectives, materials and activities. The content of the intervention is based on memory training from different perspectives, including cognitive and emotional aspects and social and individual skills. Data will be collected at baseline, at 3–4 months and at 6 months. To assess the efficacy of the intervention on cognitive function, health-related quality of life and functionality, a statistical analysis will be performed by fitting a repeated-measures mixed effects model for each main outcome: *Self-perceived memory,* measured by a Subjective Self-reported Memory Score (from 0 to 10) and by the Memory Failures in Everyday life questionnaire (MFE); *Everyday memory,* measured using the Rivermead Behavioural Memory Test-3 (RBMT-3) and *Executive control abilities,* measured in terms of visual-perceptual ability, working memory and task-switching ability with the Trail Making Test (TMT) and with the digit span scale of the Wechsler Adult Intelligence Scale III (WAIS III).

**Discussion:**

The results of this study will be highly useful for social and public health policies related to older people. Given the continuous increase in the prevalence of older people, a large number of interventions targeting memory loss are funded by public resources. To ensure transparency and effective prioritization, research such as the present study is needed to provide evidence of the effectiveness and usefulness of these interventions.

**Trial registration:**

Number: NCT02431182.

## Background

Social changes in the twentieth century have been characterized by worldwide population aging, especially in developed countries. This has led to major changes in disease distribution, with an increase in the prevalence of chronic and degenerative diseases, including cognitive impairment and dementia. Between 2000 and 2050, the proportion of the global population older than 60 years will double from approximately 11 % (605 million people) to 22 % (2 billion people) [1]. In Spain, the estimated proportion of people older than 64 years in 2050 is approximately 30 % of the population [2]. In 2012, an estimated 8.4 million people aged 60 years and older had dementia in European Union member states, accounting for 7 % of the population in that group. Spain had one of the highest prevalence rates of dementia, which affects more than 7.5 % of the population aged 60 years and older [3].

Consequently, there is increased interest in new pharmacologic treatments for dementia as well as in non-pharmacological preventive interventions. These non-pharmacological interventions are mainly based on cognitive training to optimize the performance of existing functions and to decrease the risk of cognitive impairment. The benefits of teaching rehabilitative and compensatory strategies in older persons who already have memory complaints have been shown in multiple studies [4]. However, the evidence on the effects of cognitive training on cognition in healthy elderly persons is less conclusive [5]. A systematic review of randomized controlled trials in healthy older persons concluded that training improved immediate performance on related tasks, but that there was no evidence for any generalizability of training [5]. Among the studies included in that review, a notable publication was the ACTIVE trial, which included 2800 healthy elders. In that trial, participants demonstrated significant changes in each of the three cognitive abilities for which they had received training: memory, inductive reasoning, and speed of processing. The authors estimated that the magnitude of the observed effect was equivalent to avoiding the impairment that would take place in the natural course of life of healthy people in a period between 7 and 14 years [6]. Overall, some of the characteristics associated with the success of the interventions described were the group structure, the duration (at least 8–10 sessions) and the intensity (60–90 minutes each session). In terms of content, there was no clear pattern of methods or strategies, although most of the interventions that were successful used memory training techniques, especially for episodic memory [7, 8].

Given the limited evidence available on the efficacy and social utility of cognitive training, we have designed a randomized controlled trial to assess the effectiveness of cognitive training through memory training workshops offered to healthy older persons in terms of their immediate and delayed impact on cognitive function, health-related quality of life and functionality.

The paper partially meets the requirements for the PhD program of Anna Pérez Giménez at the Pompeu Fabra University (Barcelona, Spain).

### Hypothesis

The group memory training intervention proposed in this protocol will significantly improve cognition (memory, attention and executive control abilities) and health-related quality of life (HRQoL) in the experimental group (EG) compared with the control group (CG).

### Objectives

#### Principal objective

To assess the efficacy of a memory training workshop in improving self-perceived memory, everyday memory, and executive control abilities.

#### Secondary objective

To assess the effect of the memory training workshop on HRQoL and on late-life function and disability.

## Methods

### Study design

This is a randomized controlled trial, comparing a group of individuals taking part in the memory training workshops (EG) with another group with similar characteristics not attending the workshops (CG). Data will be collected at baseline and at 3 and 6 months.

### Study setting and participants

The study will be conducted in health care centers in Barcelona (Spain). Included participants comprise healthy older people registered in any of the four health care centers included in the study and meeting the inclusion criteria. The inclusion criteria are: a) men and women aged between 65 and 80 years (inclusive) who consent to participate in the study; b) not presenting with diagnoses of dementia or cognitive impairment measured through the Mini-Mental State Examination (MMSE >24) [9, 10] and c), absence of depression measured using the Geriatric Depression Scale (GDS 5 ≤ 2) [11]. The exclusion criteria are: a) having participated in a memory training program within the last 3 years; b) having a severe sensorial disability and c) being unable to read or write. Each participant has the right to withdraw from the study at any time. In addition, a participant may be withdrawn from the study if deemed necessary by the researchers based on any of the following withdrawal criteria: a) adverse experience; b) significant non-adherence to the intervention (missing more than 50 % of the sessions); c) loss-to-follow-up; d) clinical conditions not allowing the participant to continue in the study (such as illness and accident); and e) death.

**Fig. 1:**
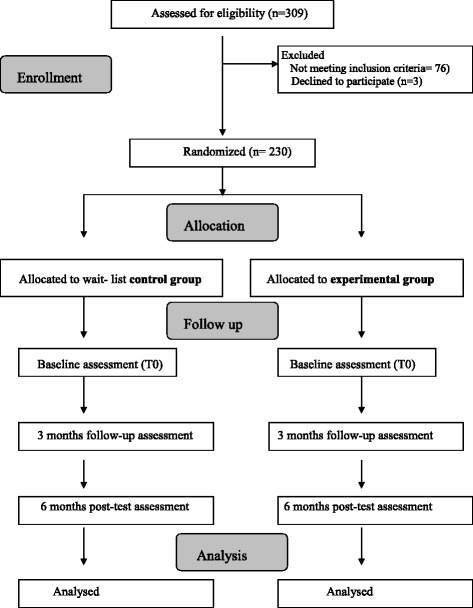
Flowchart of enrollment, allocation and follow up

Description of the study (Figure 1):

Figure 1 outlines the participant flowchart of the study procedure:

1. *Enrollment*: Recruitment will be conducted in the four participating health care centers through printed materials (leaflets and posters) in the health care centers and close community sites (library, pharmacy, market or older persons’ meeting center). Interested individuals will be required to contact their corresponding health care center, where they will first be screened for age (older than 65 years and younger than 81 years) and previous memory training (not having received memory training during the 3 previous years). Those who meet both criteria will be given an appointment to attend an inclusion interview with a study-specific trained physician or nurse at the health care center. Informed consent follows the requirements of the l’Institut d’Investigació en Atenció Primària Jordi Gol (IDIAP Jordi Gol) Clinical Research Ethics Committee. Written consent to participate will be obtained from each person at the beginning of the screening interview. Participants will be selected through the screening interview, which will be used to gather socio-demographic data and enquire about the participant’s medical history and active clinical diagnoses and medications. The GDS-5 and the MMSE will be administered.
2. *Allocation:* After the screening interview, and if the participant is eligible and willing, he or she will be randomly assigned to the EG or to the CG using a closed, opaque envelope with the group information inside. The total time required for screening will be around 30 minutes.
3. *Baseline and follow-up assessments*: Three assessments will be conducted during the study period: at baseline after randomization, between 3 and 4 months post-intervention (short-term post-test), and at 6 months post-intervention (long-term post-test). All three assessments will collect information on the variables detailed in Table 1 and will be conducted by a trained psychologist through in-person interviews in the health care center. Time requirements will be approximately 75 minutes for the baseline assessment and approximately 90 minutes for the follow-up assessments. Standardization of data collection procedures will be ensured through a variety of training and quality-control procedures adhering to CONSORT guidelines for transparent reporting [12]. Data collectors will be blind to the treatment group assignment. All data collectors will participate in an intensive 2-day training workshop that will include information on the study design, recruitment issues, and general research interviewing principles; detailed instruction on the administration of each test or measurement procedure; demonstrations of each test/measurement; and practice sessions with other data collectors. Furthermore, the data collectors will use a user-friendly answer sheet specially designed for the study. All completed sheets will be reviewed by the fieldwork coordinator who, if there are missing data or possible mistakes, will contact the data collectors to resolve any queries.

**Table 1:** Tests and other data for the evaluation of the memory training workshop

	ASSESSMENTS			
	Screening	Baseline	Immediate Post-test	Delayed post-test
Medical History	X			
Socio-demographic data	X			
GDS-5	X		X	X
MMSE	X		X	X
RBMT-3		X	X	X
SF-36		X	X	X
Subjective Self-reported Memory Score		X	X	X
MEF		X	X	X
WAIS		X	X	X
TMT		X	X	X
SF-LLFDI		X	X	X

### Description of the intervention

Once the baseline assessment has been conducted, individuals in the EG will start the multifactorial intervention. The memory training workshop is designed to stop or delay age-related memory losses and promote personal autonomy, thus enhancing mental and physical wellbeing. Specifically, these workshops aim to broaden knowledge on memory function, improve memory processes, and increase self-esteem and quality of life by providing some strategies (i.e., strategies for collecting and coding information) and relational space. The intervention will consist of twelve 90-minute group sessions provided once a week by a psychologist specialized in memory training. The groups will be composed of around 15 people. Each session will have its own objectives, materials, and activities (Table 2). The content of the intervention is based on memory training from different perspectives such as cognitive and emotional aspects and social and individual skills.

**Table 2:** Contents of the memory training workshop by session

SESSION	CONTENT
1	Presentation of the workshop: Objectives and Methodology
2	Types of memory I: Explanation of the different types of memory and their role in normal life
3	Types of memory IIa: Strategies for gathering and codifying information.
4	Types of memory IIb: Strategies for selecting and storing information.
5	Types of memory III: Strategies for gathering, codifying, selecting, storing and retrieving information. Importance of the context and the meaning of things. Semantic and episodic memory.
6	Types of memory IV: Strategies for gathering, codifying, selecting, storing and retrieving information. Individual differences.
7	Types of memory V: Strategies for retrieval according to individual profile. Improving self-esteem.
8	Types of memory VI: Strategies for recovering social skills.
9	Personal and social skills
10	New contexts and opportunities. Conceptual maps (physical and mental).
11	Evaluation of the workshop.

### Outcome measures

Screening variables: *Depressive symptoms* will be measured using the GDS [13], which has been adapted and validated for the Spanish geriatric population [14]. The shortened, GDS-5 will be used to screen potential participants [15]. The GDS-15 version will be used for the follow-up assessments [16]. *Cognitive function* will be evaluated using the 11-item MMSE, which will be used to screen probable cognitively-impaired individuals (MMSE values below 24 points) [17]. *Sociodemographic information* will be collected on age, gender, marital status, living arrangements, education level, last occupation, and residential neighborhood. Individual socioeconomic status will be obtained through the last occupation of the individual according to the last classification of the Spanish Society of Epidemiology [18]. The residential neighborhood will allow an ecological socioeconomic score to be obtained, based on indicators such as graduate rates, unemployment rates, vehicles, second-hand home prices, and the Disposable Household Income Index [19].

### Main outcomes

*Self-perceived memory* will be measured using a subjective self-reported memory score (from 0 to 10), as well as with the Memory Failures in Everyday life questionnaire (MFE). This questionnaire consists of 28 items and uses a Likert-type scale referring to perception of everyday memory problems [20–22].

*Everyday memory* will be measured using the Rivermead Behavioural Memory Test-3 (RBMT-3) [23]. This cognitive test evaluates different types of memory, such as associative memory, prospective memory, visual memory, topographical memory, and verbal memory. The test provides a General Memory Index and different subtest scales.

*Executive control abilities will be measured in terms of* visual-perceptual ability, working memory and task-switching ability with the Trail Making Test (TMT) [24]. This neuropsychological test is divided into two parts and the score on each part represents the amount of time required to complete the task [25]. Working memory will also be assessed using the digit span scale of the Wechsler Adult Intelligence Scale III (WAIS III).

### Secondary outcomes

*Health-Related Quality of Life* (HRQoL) will be assessed using the Short Form-36 (SF-36) [26, 27], with a validated Spanish version [28, 29]. Of the 36 items, 35 make up eight scales: physical functioning, role limitations due to physical functioning, bodily pain, general health perceptions, vitality, social functioning, role limitations due to emotional problems, and mental health [26].

*Functionality and disability* will be measured with the Spanish Version of the Short-Form Late-Life Function and Disability Instrument (SFLLFDI) [30, 31]. The LLFDI was developed as a comprehensive assessment of function and disability for use in community-dwelling older adults. It contains items that represent functional limitations (inability to perform discreet physical tasks encountered in daily routines) and disability (inability to take part in major life tasks and social roles).

### Intervention-related variables

Other variables related to the implementation of the intervention will be collected, such as the number of sessions the person has attended, the participants’ satisfaction with the workshops, the usefulness of the program, and overall impressions. The professionals conducting the workshops will also evaluate individual and group progress.

### Sample size calculation

The sample size was calculated to detect a difference in everyday memory higher than 2 points (0.5 SD) on the mean RBMT-3 score between the control and the intervention group [32]. Accepting an alpha risk of 0.05 and a beta risk of 0.1 in a bilateral contrast, and assuming a mean score of 16.07 and an SD of 4.19 [33] on the RBMT-3 in the Spanish population aged between 70 and 75 years, 75 individuals in each group will be needed. To ensure this sample size and considering an estimated rate of losses of 20 % in the EG and 40 % in the CG, the final sample size needed is 195 individuals (90 in EG and 105 people in CG, respectively).

### Statistical analysis

Baseline comparability between the study arms will be assessed through bivariate analyses of the pre-test data set group (dependent and independent variables) comparing the control and the experimental arms. The chi-square test will be used to compare qualitative data, as well as the Student *t* test or the Mann–Whitney test for quantitative data, depending on the normality of the distribution. If differences are found in baseline characteristics, we will consider adjusting for these variables in the efficacy analysis.

A descriptive analysis will be conducted for results at baseline, at the immediate follow-up and at the delayed follow-up for the EG and CG. Categorical outcomes will be described through raw frequencies or percentages, and continuous outcomes will be described through mean scores and standard deviations. Effect measures for each outcome at the immediate and delayed follow-ups will be computed as the mean of the differences of the aggregated scores for the EG and CG. Effect measures will be presented with their 95 % confidence intervals.

The main statistical analyses to assess the efficacy of the intervention on self-perceived memory, everyday memory and executive control abilities will be performed using a repeated-measures mixed effects model for each main outcome defined in the protocol [34]. In each model, the independent variables will be the group (EG or CG), time (baseline, immediate follow-up or delayed follow-up) and any relevant characteristics that differ between the groups at the baseline assessment (e.g., age or educational level). An interaction term between time and group will be included in the analyses. The models will be fitted with the available data, ignoring missing data.

To determine whether the results have been influenced by missing data, we will conduct sensitivity analyses by fitting the models with completed datasets obtained by imputing the missing data [35]. In this analysis, missing values for the immediate or delayed post-test assessments will be imputed through the Last Observation Carried Forward (LOCF) strategy. According to this strategy, missing values will receive the value obtained by the participant in the previous assessment. In addition, we will perform sensitivity analyses restricted to those participants attending at least 80 % of the sessions.

### Ethics considerations

The participants will be informed both verbally and in writing about the aims, methods, procedures and measures performed during this study. They will also be informed about ethical issues such as confidentiality, their right to ask any questions during the study and their right to withdraw at any time. To ensure that all participants have received information about this research project and agreed to participate, all participants will be asked to sign a written consent form. The research team is committed to performing this study according to the Good Clinical Practice Guidelines of the Declaration of Helsinki. This protocol was approved by the Comitè Ètic d’Investigació Clínica (CEIC) del IDIAP Jordi Gol.

## Discussion

### Quality control

Reliability and validity are considered to be the key criteria for assessing the quality of quantitative studies. To assure these qualities, the randomization list of this randomized controlled trial will be computer-generated and will remain concealed until the participants’ assignment to each group. After the informed consent form has been signed, a study entry number will be assigned to each participant. This number will be on the outside of a closed, opaque envelope, which will allocate the participant to either the EG or the CG (single-blind allocation). Allocation concealment will prevent the occurrence of selection bias before the interventions are provided to the participants [36]. In addition, the professionals who will carry out the neurocognitive evaluations (pre-test, short-term post-test and long-term post-test) and the memory training workshop professionals will not be aware of the group to which each participant belongs.

The following procedures will also be used to guarantee the quality of the information. The intervention will be carried out by trained personnel who will follow the same protocol and materials, thus guaranteeing the homogeneity of the intervention. The same will occur with data collection, which will be conducted by specially trained professionals. Data will be collected through a specifically created data-register sheet designed by Teleform® v.10.2 software. This automated software reads scanned questionnaires and performs a validation process of the data with previously established parameters; once the data have been validated, they are entered into a specifically created dataset.

### Implications

The memory training workshop proposed in the present study could be considered as paradigmatic of the memory training interventions that are being conducted in Barcelona, even in Spain, as most of them follow the same pattern. [8, 32, 37–39]. The results of this study will be very useful for social and public health policies for older people. The findings of this study should be considered when decisions need to be made regarding investing public resources in certain interventions.

### Strengths and limitations

Our study has some important strengths; it is the first randomized controlled trial aiming to demonstrate the effectiveness of memory training in healthy older people in Spain. Additionally, we will apply a wide variety of validated tests, which will allow for assessment of the potential impact of the intervention on several health-related outcomes (memory, other executive control abilities, HRQoL, life function and disability), as well as comparisons with other studies.

A possible limitation is the length of follow up, less than 1 year, which may negatively affect our results because some increases in memory problems may not be detected. Another limitation of our study is that it does not include a matched active control group. Therefore, we will not be able to isolate the memory training effects from the potential confounding benefits of increased social contact.

## Abbreviations

CG: Control Group
EG: Experimental Group
GDS: Geriatric depression scale
HRQoL: Health-related quality of life
MMSE: Mini-mental state examination
MFE: The memory failures of everyday questionnaire
RBMT-3: Rivermead behavioural memory test-3
RCT: Randomized controlled trial
SFLLFDI: Spanish version of the short-form late-life function and disability instrument
SD: Standard deviation
TMT: Trail making test
WAIS: Wechsler adult intelligence scale
